# Sphere-forming culture enriches liver cancer stem cells and reveals Stearoyl-CoA desaturase 1 as a potential therapeutic target

**DOI:** 10.1186/s12885-019-5963-z

**Published:** 2019-08-01

**Authors:** Xiao-Lu Ma, Yun-Fan Sun, Bei-Li Wang, Min-Na Shen, Yan Zhou, Jian-Wen Chen, Bo Hu, Zi-Jun Gong, Xin Zhang, Ya Cao, Bai-shen Pan, Jian Zhou, Jia Fan, Wei Guo, Xin-Rong Yang

**Affiliations:** 1 Department of Laboratory Medicine, Zhongshan Hospital, Fudan University, 136 Yi Xue Yuan Road, Shanghai, 200032 People’s Republic of China; 2 Department of Liver Surgery, Liver Cancer Institute, Zhongshan Hospital, Fudan University, Key Laboratory of Carcinogenesis and Cancer Invasion, Ministry of Education, 136 Yi Xue Yuan Road, Shanghai, 200032 People’s Republic of China; 3 Cancer Research Institute, Xiangya School of Medicine, Central South University, Key Laboratory of Carcinogenesis and Cancer Invasion, Ministry of Education, Changsha, 410078 China

**Keywords:** Cancer stem cell, Hepatocellular carcinoma, Stearoyl-CoA desaturase 1, Sphere-forming assay

## Abstract

**Backgrounds:**

The role of sphere-forming culture in enriching subpopulations with stem-cell properties in hepatocellular carcinoma (HCC) is unclear. The present study investigates its value in enriching cancer stem cells (CSCs) subpopulations and the mechanism by which HCC CSCs are maintained.

**Methods:**

HCC cell lines and fresh primary tumor cells were cultured in serum-free and ultra-low attachment conditions to allow formation of HCC spheres. In vitro and in vivo experiments were performed to evaluate CSC characteristics. Expression levels of CSC-related genes were assessed by qRT-PCR and the correlation between sphere formation and clinical characteristics was investigated. Finally, gene expression profiling was performed to explore the molecular mechanism underlying HCC CSC maintenance.

**Results:**

We found that both cell lines and primary tumor cells formed spheres. HCC spheres possessed the capacity for self-renewal, proliferation, drug resistance, and contained different subpopulations of CSCs. Of interest, 500 sphere-forming Huh7 cells or 200 primary tumor cells could generate tumors in immunodeficient animals. Sphere formation correlated with size, multiple tumors, satellite lesions, and advanced stage. Further investigation identified that the PPARα-SCD1 axis plays an important role in maintenance of the CSC properties of HCC sphere cells by promoting nuclear accumulation of β-Catenin. Inhibition of SCD1 interfered with sphere formation, down-regulated expression of CSC-related markers, and reduced β-Catenin nuclear accumulation.

**Conclusions:**

Sphere-forming culture can effectively enrich subpopulations with stem-cell properties, which are maintained through activation of the PPARα-SCD1 axis. Therefore, we suggest that targeting the SCD1-related CSC machinery might provide a novel insight into HCC treatment.

**Electronic supplementary material:**

The online version of this article (10.1186/s12885-019-5963-z) contains supplementary material, which is available to authorized users.

## Background

Hepatocellular carcinoma (HCC) is the fifth most prevalent malignancies in the world and third most frequent cause of cancer death [1]. Currently, surgery remains the most effective treatment with curative potential; however, only about 10–20% of patients with HCC are eligible for surgical intervention [2–4]. Meanwhile, more than 50% patients will have tumor relapse and metastasis during the five years following curative resection [4]. Thus, a better understand of HCC biology and behavior will lead to advances in treatment.

The cancer stem cell (CSC) hypothesis posits that a small subset of cancer cells, with stem cell-like properties, has the capacity to induce tumor recurrence or metastasis, driving tumor progression and resistance to traditional therapies [5–7]. Targeted therapies aimed at eradicating CSCs might lead to the development of more effective treatment strategies [8]. In HCC, CSCs were first defined as a side population [9]. However, isolation of the side population using Hoechst dye staining may not accurately identify the real CSC population, due to an artifact of Hoechst 33342 toxicity rather than their intrinsic stem-cell properties.

Recently, HCC CSCs were identified based on the expression of various cell surface markers, including CD90 [10], CD13 [11], CD133 [12], epithelial cell adhesion molecular (EpCAM) [13], CD24 [14], OV6 [15], and Intercellular adhesion molecule 1 (ICAM1) [16]. However, these markers are not expressed exclusively in liver CSCs and their distribution in HCC is heterogeneous. Given the lack of HCC CSC-specific biomarkers, we aimed to develop alternative methods for isolating HCC CSCs.

The sphere-forming assay was first introduced as a functional approach for studying adult stem cells [17] and has been widely used to evaluate the stem properties of proposed CSC populations [18–23]. Using anchorage-independent sphere culture with serum-free, non-adherent, and nutritionally deficient conditions, differentiated tumor cells undergo apoptosis, while CSCs survive, adapt, and proliferate [17, 24]. This experimental approach is based on inherent characteristics of CSCs, it can enrich relatively whole subpopulations of CSCs regardless of their expression patterns. Sphere culture approach thus represents an optimal method for enriching CSC subpopulations from whole tumors. Recently, sphere formation has been used to enrich the potential CSC subpopulation in HCC cell lines [25, 26]. However, there are currently no published data that comprehensively demonstrate the CSC properties of HCC sphere cells, particularly those derived from primary patient tissues.

In this study, we enriched the CSC subpopulation from HCC tumor tissues and cell lines using sphere culture, and evaluated the differential expression profiles of tumor sphere and parental cells to explore the potential mechanism of CSC maintenance. We found that sphere-forming culture effectively enriched the HCC CSC subpopulation and promotes CSC properties via activation of the peroxisome proliferator-activated receptor-alpha (PPARα)-stearoyl-CoA desaturase (SCD1) axis. We suggest that targeting the SCD1 signaling pathway might be a novel therapeutic approach for the treatment of HCC.

## Methods and materials

### Cell lines and cell culture

Huh7 and Hep3B cell lines were provided by the Cell Bank at the Institute of Biochemistry and Cell Biology, China Academy of Science (Shanghai, China). Both cell lines were cultured in Dulbecco’s modified Eagle’s medium (DMEM) containing 10% fetal bovine serum (FBS), supplemented with 100 IU/ml penicillin and 100 μg/ml streptomycin, and incubated at 37 °C in a humidified atmosphere with 5% CO_2_. All cell culture reagents were obtained from Gibco (Invitrogen, USA).

### Sphere-forming assay

Serum-free medium for sphere culture was composed of DMEM/F12 medium supplemented with 100 IU/ml penicillin, 100 μg/ml streptomycin, 20 ng/ml human recombinant epidermal growth factor, 20 ng/ml human recombinant basic fibroblast growth factor, 1% nonessential amino acids, 1% GlutaMax, 2% B27 supplement (Invitrogen, USA), and 1% methylcellulose (Sigma, USA). HCC cells were cultured at a density of 1000 cells/ml; when spheres reached a diameter of 100 μm, the sphere-forming efficiency was calculated and spheres were collected for further use [13].

### Immunofluorescent staining

Spheres were fixed in 4% paraformaldehyde and blocked with 5% bovine serum albumin. Antibodies, including phycoerythrin (PE)-conjugated mouse anti-human CD133 and PE-conjugated mouse anti-human EpCAM (both 1:50, MiltenyBiotec, Germany) were added and incubated overnight at 4 °C. After washing with phosphate-buffered saline three times, spheres were counterstained with DAPI (Sigma-Aldrich, USA). For β-Catenin staining, 0.1% Triton was used for permeabilization. After blocking, mouse anti-human β-Catenin antibodies (BioLegend, 1:30) were added and incubated overnight at 4 °C. Sphere cells were also counterstained with DAPI (Sigma-Aldrich, USA). Images were captured using an IX-71 fluorescent microscope (Olympus, Japan).

### Colony formation assay

Once they reached a diameter of 100 μm, HCC spheres were collected through gentle centrifugation, dissociated with trypsin-EDTA (Invitrogen, USA), and mechanically disrupted with a pipette. The resulting cells were gently centrifuged to remove trypsin. Single cells were seeded in DMEM with 10% FBS (Gibco, USA) at a density of 2000 cells per well in a 6-well plate (Corning, USA). Parental Huh7 cells were seeded at the same density as a control population to evaluate colony-forming capacity. After two weeks, the colony-forming ability was assessed by counting the number of colonies (> 70 cells) under a microscope after staining with crystal violet (Sigma-Aldrich, USA). Representative images were photographed using an Olympus LX-71 fluorescence microscope. Experiments were performed in triplicate.

### In vitro differentiation assay

Hep3B and Huh7 cells were grown in serum-free conditions to induce initial sphere formation, then 10% FBS was added to induce HCC sphere differentiation. FBS was removed and the first differentiated sphere cells were grown in serum-free conditions again to induce the second sphere formation. 10% FBS was added to induce differentiation of the second HCC spheres. This process was performed once more to generate three rounds of spheres and differentiated cells. The three sets of HCC spheres and differentiated sphere cells were harvested and RNA was extracted for PCR analysis.

### RNA extraction and quantitative RT-PCR (qRT-PCR)

Total cellular RNA extraction was performed using a RNeasy mini kit (Qiagen, Germany) and cDNA was synthesized using the Quantitect Reverse Transcription Kit (Qiagen, Germany) according to the manufacturer’s instructions. Target genes were quantified using FastStart Universal SYBR Green Master (Roche diagnostics, Germany) and DNA amplification was carried out using a LightCycler 480 (Roche Diagnostics, Germany). The relative quantities of target gene mRNAs compared to an internal control were determined using the ΔCq method. PCR conditions were as follows: 5 min at 95 °C, followed by 40 cycles of 95 °C for 10 s and 60 °C for 60 s. GAPDH was used as an internal control. Primers and probes are listed in Additional file 1: Table S1.

### Drug treatment

The sensitivity of normal HCC cells and sphere HCC cells to chemotherapeutic drugs were measured using a Cell Counting Kit-8 (CCK-8) assay (Dojino, Japan). Cells were seeded at a density of 1 × 10^3^ of cells in 96-well plates, and were incubated with 80 mM 5-Fluorouracil (5-FU, Sigma, USA), 5 μmol/L Sorafenib (MCE, USA), or 2 μmol/L Doxorubicin (MCE, USA) for 48, 72, and 96 h. All experiments were performed in triplicate. CCK-8 reagent was then added to each well according to the manufacturer’s instructions. For PPARα signaling inhibition, Huh7 and Hep3B cells were treated with 25 μM GW6471 (Sigma, USA), a PPARα inhibitor, for 48 h. For SCD1 inhibition, Huh7 and Hep3B cells were treated with 20 μM PluriSIn #1 for 48 h.

### Fresh clinical tissue specimens

Twenty-five fresh HCC tissue samples were collected from patients at Zhongshan Hospital in September 2013. These patients received no previous local or systemic treatment before resection. Surgical specimens were obtained at the time of resection from all patients. All samples were received in the laboratory within one hour, immediately mechanically disaggregated and digested with type IV collagenase (Gibco, USA), and re-suspended in DMEM medium. Single-cell suspensions were obtained by filtration through a 40 μm filter. Red blood cells were lysed with ACK buffer (Invitrogen, USA). The number of viable cells was counted and analyzed using Trypan blue. Isolated primary cells were then cultured in serum-free medium at a density of 20,000/well in an ultra-low attachment 6-well plate [11]. Approval for the use of human subjects was obtained from the research ethics committee of Zhongshan Hospital. Written informed consent was obtained from each subject enrolled in this study.

### Tumorigenicity experiments

In our study, tumorigenicity was defined as the capacity of a certain cell number, following serial dilution, to form tumor nodules in immunodeficient mice within a certain time interval. Six-to-eight-week-old male NOD/SCID mice were randomly divided into groups (six mice/group) and maintained under standard conditions, according to institutional guidelines. Cells were suspended in a serum-free DMEM/Matrigel (BD Biosciences, USA) mixture (1,1 by volume), and injected subcutaneously into the flanks of recipient NOD/SCID mice. Tumor formation was monitored every two weeks following injection, and the size and incidence of tumors were recorded. The tumorigenicity experiment was terminated six weeks after injection, at which point mice with no apparent tumor nodules at the injection site were considered negative.

### cDNA microarray

cDNA expression profiling was performed using total RNA with the GeneChip Human Genome U133 Plus 2.0 Array (Affymetrix, USA) according to the manufacturer’s instructions and a previous report [27].

### Statistical analysis

Statistical analyses were performed using SPSS 20.0 software (IBM, Chicago, IL, USA). Experimental values for continuous variables were expressed as the mean ± standard error of the mean. The chi-squared test, Fisher’s exact probability tests, and the Student’s *t-*test were used as appropriate to evaluate the significance of differences in data between groups. If variances within groups were not homogeneous, a nonparametric Mann–Whitney test or a Wilcoxon signed-rank test was used. The relationships between sphere-formation capacity and TTR were analyzed using Kaplan–Meier survival curves and log-rank tests, respectively. A *p* value < 0.05 was considered statistically significant.

## Results

### HCC cell lines form spheres with CSC properties

**Fig. 1:**
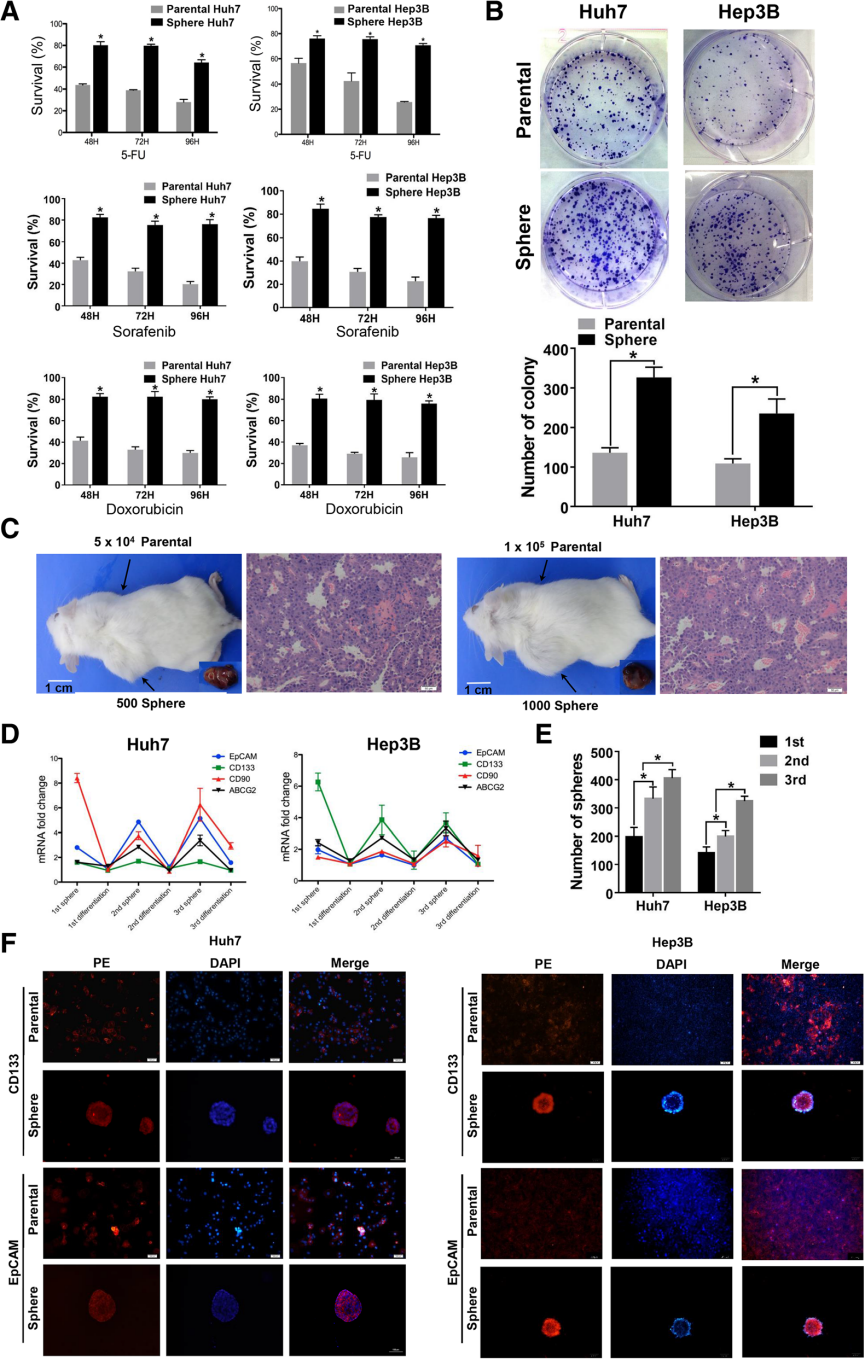
Cancer stem cell (CSC) properties of sphere cells in HCC cell lines. **a** Survival rates of Huh7 (left) and Hep3B (right) after 80 μM 5-FU (upper), 5 μM Sorafenib (middle), or 2 μM Doxorubicin (lower) treatment were evaluated by CCK8 assay. **b** Representative photographs of the plates containing colonies derived from 2000 sphere or parental normal Huh7 (upper) and Hep3B (lower) cells. Colony formation experiments were performed in triplicate (mean ± SD). **c** Representative NOD/SCID mice with subcutaneous tumors from sphere Huh7 cells and H&E staining of subcutaneous nodules. Scale bar 1 cm. **d** Expression levels of EpCAM, CD133, ATP-binding cassette sub-family G member 2 (ABCG2) and CD90 among the 1st, 2nd, 3rd sphere and differentiated sphere cells in Huh7 (left) and Hep3B (right) cells. Results were normalized according to the expression of parental cells. All experiments were done in triplicate. **e** Evaluation of sphere formation rates in three sequential generations of Huh7 and Hep3B cells. **f** Expression of epithelial cell adhesion molecule (EpCAM) and CD133 in 2nd sphere and parental normal Huh7 (left) and Hep3B (right) cells. Scale bar 100 μm

Two HCC cell lines (Huh7 and Hep3B) were cultured in ultra-low attachment surface plates with serum-free medium, and both cell lines formed sphere clusters. As drug resistance is a main characteristic of CSCs, we treated sphere-forming cells with 5-FU, Sorafenib, or Doxorubicin to evaluate drug resistance. We found that the sphere-forming cells of both cell lines had greater tolerance to treatment with a high concentration of 5-FU (80 mmol/L), Sorafenib (5 μmol/L) and Doxorubicin (2 μmol/L) than their corresponding parental cells (Fig. 1a). These results suggest that these sphere-forming subgroup cells may have a survival advantage when exposed to cytotoxic drugs.

We also evaluated the colony-forming capabilities of HCC sphere cells, and found that the sphere cells proliferated significantly faster and formed bigger colonies than parental cells after three weeks of culture. We observed a greater number of colonies following seeding of 2000 cells in tumor sphere cell cultures compared with parental cells (Huh7 307.33 ± 29.00 vs. 148.33 ± 19.43, Hep3B 235.66 ± 14.85 vs. 97.67 ± 6.06; both *p* < 0.05) (Fig. 1b).

In vivo serial dilution tumorigenesis assays are considered to be the golden standard for evaluating CSC properties; therefore, parental and sphere Huh7 cells were transplanted in NOD/SCID mice. We found that as few as 500 Huh7 sphere cells were sufficient for tumor development, whereas, as many as 10^5^ parental Huh7 cells were unable to initiate tumor in immunodeficient mice after four weeks. The same results were observed when 1000 cells were injected, resulting in a shorter tumor formation time (less than two weeks) (Fig. 1c, Table 1, Additional file 2: Table S2). These data confirm that HCC sphere cells have efficient tumor-initiating capacity.

**Table 1:** Comparison of Tumorigenic Capacity of Sphere-forming and Normal Cultured Huh7 Cells

		No. of Mice with Tumor Formation/Total No. of Mice with Cell Injection		
Phonotypes	No. of cells injected	2 Weeks	4 Weeks	6 Weeks
Sphere-forming cells	5 × 10^2^	2/6	4/6	4/6
	1 × 10^3^	4/6	6/6	6/6
Normal cultured cells	1 × 10^4^	0/6	0/6	0/6
	1 × 10^5^	0/6	0/6	0/6

The self-renewal potential of HCC sphere cells was also evaluated using three rounds of serial passaging (see Methods). Two days after adding 10% FBS, sphere cells attached onto plates and grew as adherent cells. We compared the expression of *EPCAM*, *PROM-1* (*CD133*), *ATP-binding cassette sub-family G member 2* (*ABCG2*), and *THY1* (*CD90*) between the three sets of differentiated spheres by qRT-PCR. We found that expression of these genes was significantly higher when cells formed spheres. However, following addition of 10% FBS, spheres differentiated and the expression of these four stem cell markers decreased to the level of parental cells. Remarkably, these results were observed in three sequential generations (Fig. 1d). To further explore whether sphere formation rate increase over time, we observed the sphere formation rates in three sequential generations. Result showed significant increases of sphere formation number after every passage (Fig. 1e), indicating sphere formation percentage were escalating during serial passage. Furthermore, to confirm the qRT-PCR results, protein expression of EpCAM and CD133 in the third sphere generation and in differentiated sphere cells was evaluated with PE-conjugated anti-EpCAM/anti-CD133 antibodies (Fig. 1f).

### Evaluation of sphere cells as CSCs in human HCC clinical specimens

**Fig. 2:**
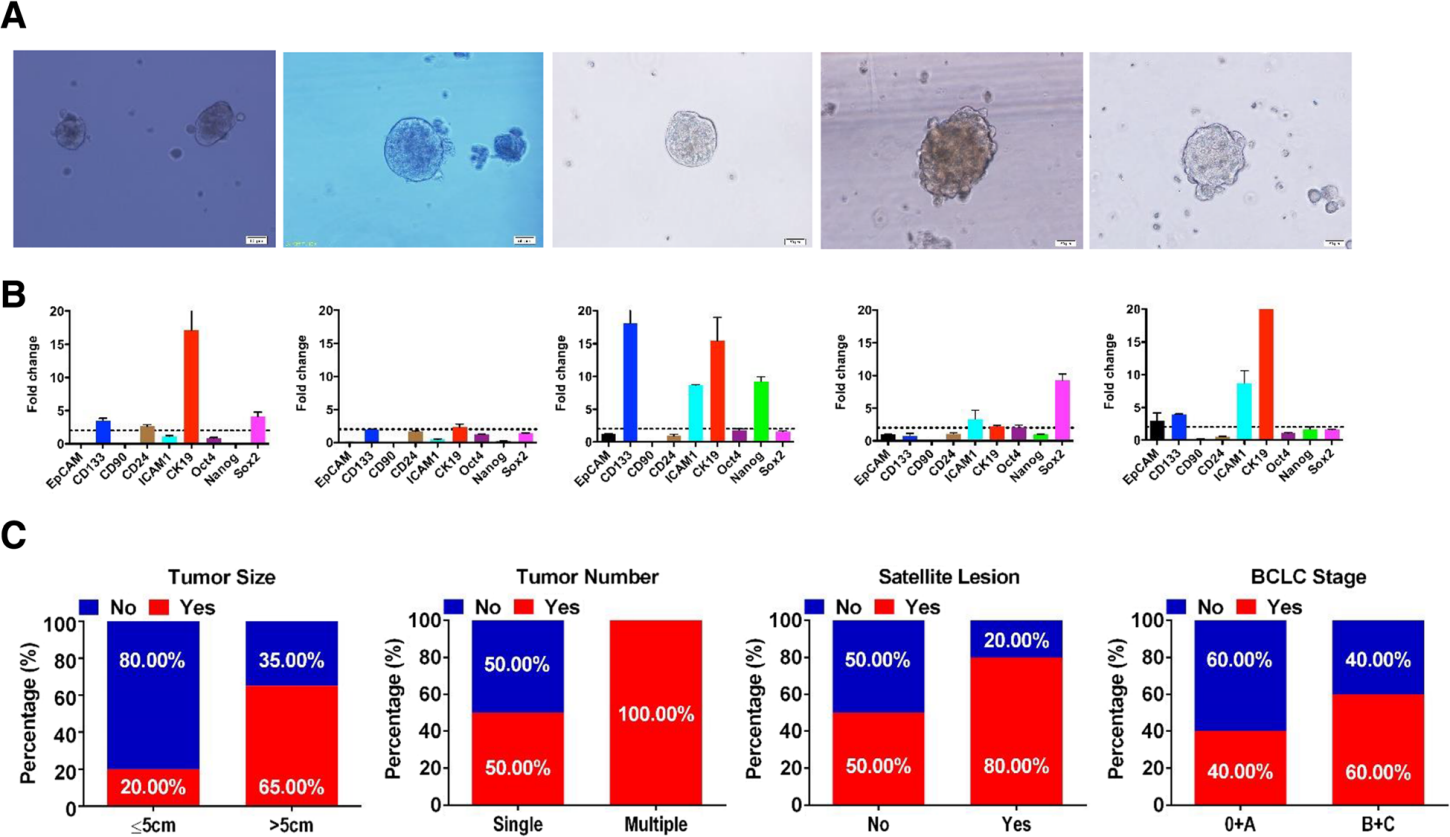
CSC properties of sphere cells in fresh clinical HCC specimens and association between sphere formation and prognosis. **a** Representative photographs of spheres formed from 5 fresh clinical HCC specimens. Scale bar 50 μm. **b** Relative expression of CSC-related genes in 5 primary HCC spheres. The expression level of certain gene in sphere cells was normalized according to the expression of that in parental HCC cells. **c** Positive rates of sphere-formation in 25 patients stratified according to tumor size, number, satellite lesion, and tumor stage

To investigate the CSC traits of sphere cells derived from fresh clinical specimens, we first successfully generated 5 cases of primary HCC spheres from 9 patients. Typical images of primary HCC spheres were shown in Fig. 2a. We next evaluated the expression of CSC markers (*EPCAM*, *PROM-1*, *THY1*, *CD24*, *ICAM1*, *KRT19*, *OCT4*, *NANOG*, and *SOX2*) in these five paired samples (sphere and corresponding parental cells) by qRT-PCR and found that different marker expression patterns occurred in different primary tumor spheres (Fig. 2b). To further explore the CSC potential and tumorigenic capability of enriched sphere tumor cells, we injected primary sphere tumor cells and corresponding tumor cells from the one randomly selected patient into NOD/SCID mice. We observed a significant difference in tumor incidence between these two cell populations: as few as 200 primary sphere cells were sufficient for consistent tumor development in immunodeficient mice, while up to 10^6^ parental tumor cells could not induce tumor formation (Table 2).

**Table 2:** Comparison of Tumorigenic Capacity of Primary Sphere Tumor cells and Primary CD45^−-^Tumor cells

		No. of Mice with Tumor Formation/Total No. of Mice with Cell Injection		
Phonotypes	No. of cells injected	2 Weeks	4 Weeks	6 Weeks
Sphere cells	2 × 10^2^	0/6	3/6	3/6
	5 × 10^2^	0/6	4/6	4/6
	1 × 10^3^	1/6	3/6	3/6
	5 × 10^3^	2/6	5/6	5/6
CD45^−^ cells	1 × 10^3^	0/6	0/6	0/6
	1 × 10^5^	0/6	0/6	0/6
	1 × 10^6^	0/6	0/6	0/6

We further evaluated the correlation between tumor sphere formation and tumor malignancy. A total of 25 HCC patients including previous 9 patients were recruited, and 56% (14/25) of which formed tumor spheres. We found that tissues from patients with larger tumors (65.00% vs. 20.00%), multiple lesions (100.00% vs. 50.00%), satellite lesions (80.00% vs. 50.00%), or advanced tumor stage (60.00% vs. 40.00%), had more efficient sphere-forming capacity under serum-free conditions (Fig. 2d).

### The role of the PPARα signaling pathway and SCD1 in maintaining stem characteristics of sphere cells

**Fig. 3:**
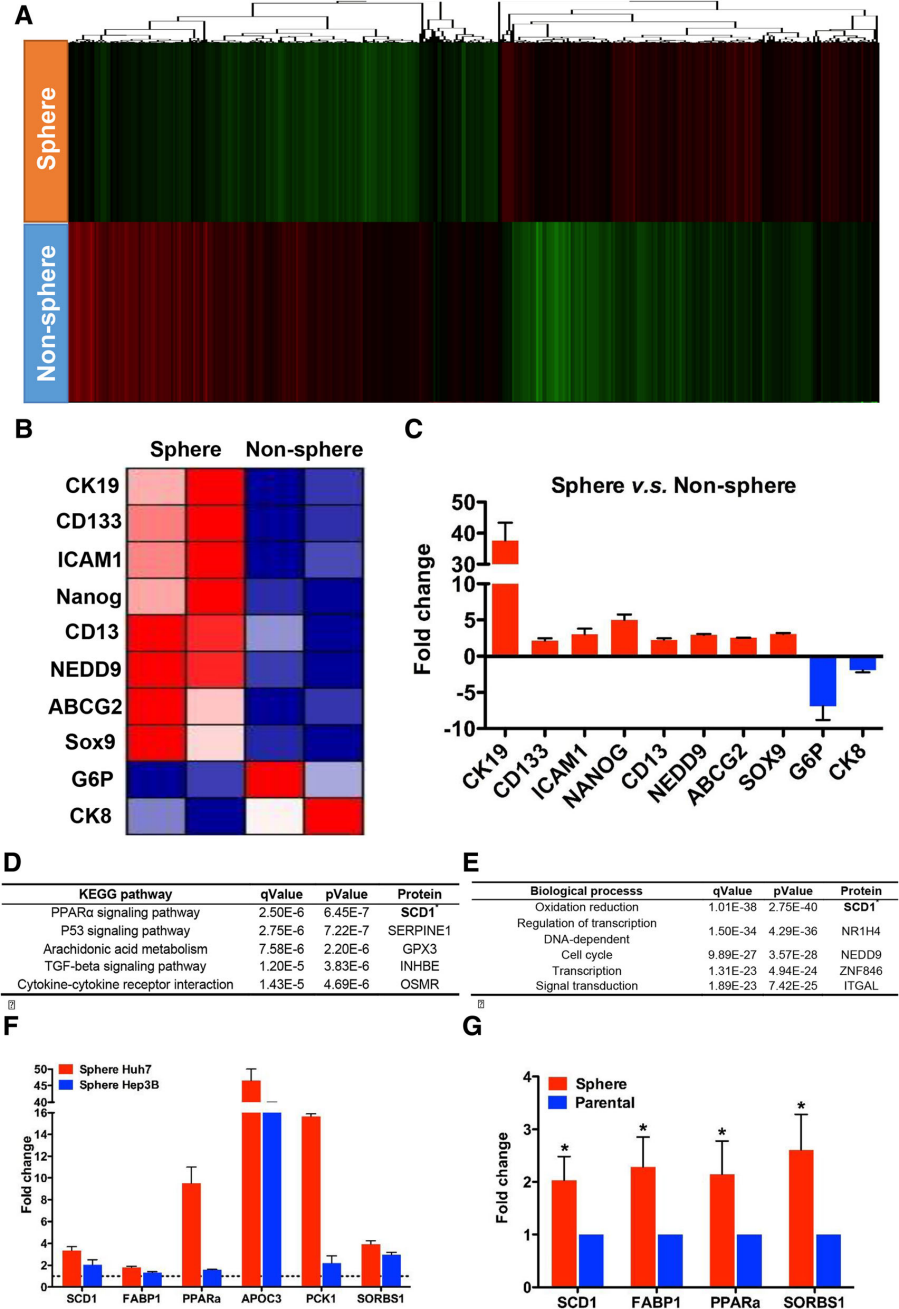
Expression profiling revealed PPARα signaling and SCD1 might contribute to CSC traits of HCC. **a** Hierarchical cluster analysis based on sphere and parental Huh7 cells. Red and green cells depict high and low expression levels, respectively. **b** Heat map of CSC-related and mature hepatocyte-related genes according to expression profile. Red and blue cells depict high and low expression levels, respectively. **c** qRT-PCR evaluation of CSC-related and mature hepatocyte-related genes. Red and blue columns depict high and low expression fold changes, respectively. **d** KEGG pathway analysis of expression profile. **e** Biological process analysis of expression profile. **f** qRT-PCR evaluation of key genes involved in PPARα pathway in Huh7 and Hep3B cell lines. **g** qRT-PCR evaluation of key genes involved in PPARα pathway in 5 primary tumor spheres. *: *P* < 0.05

To identify the potential mechanism underlying the maintenance of stem-cell phenotypes of sphere-forming cells, a microarray analysis was performed to compare the different expression profiles between Huh7 sphere and parental cells. Using a foldchange of 2.0 as the cutoff, we identified 1844 up-regulated and 2386 down-regulated genes in sphere cells compared with parental cells; a cluster analysis demonstrated the discrete nature of these two cell types (Fig. 3a). Notably, several stem-cell markers including *PROM-1* (*CD133*), *KRT19*, *ABCG2*, *CD13*, *NEDD9*, *NANOG*, *SOX9*, and *ICAM1* were up-regulated in sphere cells, while mature hepatocyte markers, such as *glucose-6-phosphatase* (*G6PC*) and *cytokeratin 8* (*KRT8*) were down-regulated in sphere cells (Fig. 3b). These findings were confirmed by qRT-PCR (Fig. 3c).

We performed further bioinformatic analyses, and found that the PPARα signaling pathway was the most significantly activated pathway according to KEGG pathway analysis. In addition, oxidation-reduction was the most significant biological process according to gene ontology analysis. Of note, SCD1 was the most up-regulated molecule involved in both the PPARα pathway and oxidation-reduction process, which suggested that SCD1 might be a key molecule involved in maintaining stem-cell phenotypes of sphere-forming tumor cells (Fig. 3d, e).

We further evaluated the expression of SCD1 and several other PPARα pathway-related genes. Compared with parental cells, the expression of key genes (*SCD1*, *FABP1*, *PPARA*, *APOC3*, *PCK1*, and *SORBS1*) involved in the PPARα pathway were significantly higher in sphere cells (Fig. 3f). Moreover, the expression of four genes (*SCD1*, *FABP1*, *PPARA*, and *SORBS1*) was significantly higher in primary sphere cells (Fig. 3g). Based on these data, we therefore speculated that the PPARα-SCD1 axis might play an important role in maintaining CSC properties of HCC sphere cells.

### Inhibition of the PPARα pathway or SCD1 induces loss of CSC properties

**Fig. 4:**
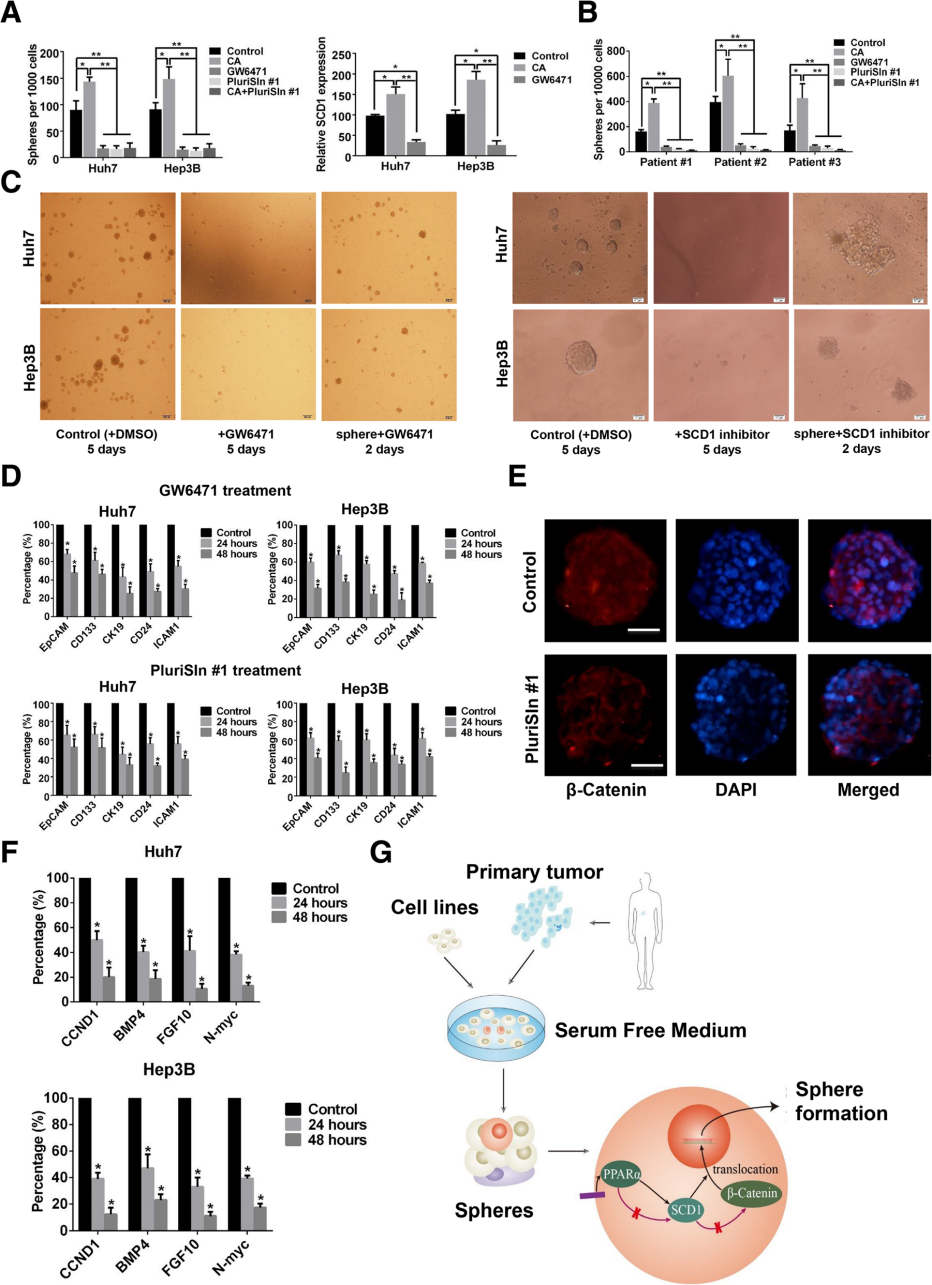
PPARα-SCD1 axis maintained CSC properties of spheres via promoting nuclear accumulation of β-Catenin. **a** Number of spheres derived from 1000 HCC cells which were treated with GW6471, PluriSln #1, or combination of clofibric acid (CA) and PluriSln #1 (left), and Relative expression of SCD1 in sphere cells after PPARα inhibition. **b** Number of spheres derived from 10000 primary HCC cells which were treated with GW6471, PluriSln #1, or combination of CA and PluriSln #. **c** Lefr panel: Representative photographs of parental HCC cells treated with DMSO for 5 days as controls, or parental HCC cells treated with 25 μM GW6471 for 5 days, or sphere HCC cells treated with 25 μM GW6471 for 2 days. Right panel: Representative photographs of parental HCC cells treated with DMSO for 5 days as controls, or parental HCC cells treated with 20 μM PluriSIn #1 for 5 days, or sphere HCC cells treated with 20 μM PluriSIn #1 for 2 days. **d** Fold changes of CSC-related markers of HCC sphere cells after treated with GW6471 (upper) or PluriSln #1 (lower) for 2 days. Results were normalized according to the expression of control spheres cells. **e** Representative immunofluorescence images of a Huh7 sphere co-stained with anti-β-Catenin and DAPI without (upper panel) or with (lower panel) SCD1 inhibition. **f** Fold changes of target genes of β-Catenin of Huh7 (upper) and Hep3B (lower) after treatment with PluriSln #1 for 2 days. Results were normalized according to the expression of control spheres cells. **g** Simplified diagram of present study. *: *P* < 0.05; ^**^: *P* < 0.001

To validate our speculation, a specific antagonist (GW6471) was used to inhibit the PPARα pathway to evaluate the role of PPARα and SCD1 in maintaining CSC properties [15]. We found that GW6471 treatment effectively decreased the sphere-forming capacity of parental Huh7 and Hep3B cells. Furthermore, treatment of parental HCC cells with a novel SCD1 inhibitor (PluriSIn #1) also decreased the sphere-forming capacity of Huh7 and Hep3B cells. To further confirm these results, we treated parental Huh7 and Hep3B cells with clofibric acid (CA), a PPARα pathway agonist, and PluriSIn #1. We found that CA could improve the sphere-formation capacity of HCC cells, while PluriSIn #1 could abolish the effect induced by PPARα activation. Moreover, qRT-PCR analysis confirmed that *SCD1* served as a functional downstream factor of PPARα as its expression significantly decreased after GW6471 treatment (Fig. 4a). We further treated primary spheres from 3 fresh specimens with CA, or PluriSIn #1, or combination of CA and PluriSIn #1. We found the results were similar to those of cell lines (Fig. 4b). Additionally, GW6471 or PluriSIn #1 treatment of HCC sphere cells not only resulted in the inhibition of sphere formation, but also could lead to gradual disintegration of spheres derived from HCC cells (Fig. 4c). Downregulation of several stem-cell markers, including *EPCAM*, *PROM-1* (*CD133*), *KRT19*, *CD24*, and *ICAM1* was observed after GW6471 or PluriSIn #1 treatment in HCC cell lines (Fig. 4d). Taken together, these data implied the vital role of the PPARα-SCD1 axis in maintaining stem properties of HCC CSC cells, and demonstrate that inhibition of SCD1 might be a promising strategy to inhibit CSCs in HCC.

SCD1 plays a role in regulating Wnt/β-Catenin signaling, which is important for maintaining CSC properties. Using immunofluorescence staining, we observed that the expression pattern of β-Catenin in most Huh7 sphere cells was nuclear; however, after short-term (24 h) SCD1 inhibition, the expression pattern became membranous (Fig. 4e). To validate these findings, expression levels of four canonical down-stream targets of β-Catenin (*CCND1*, *FGF10*, *MYCN*, and *BMP4*) were evaluated by qRT-PCR. As expected, we found that expression of all four targets was dramatically decreased after SCD1 inhibition, indicating that β-Catenin transcriptional activity was hindered by SCD1 inhibition (Fig. 4f). Collectively, our data suggest that SCD1 serves as a vital regulator of CSC maintenance in HCC via stabilization of β-Catenin transcriptional activity (Fig. 4g).

## Discussion

The identification of tumorigenic liver CSCs could provide new insights into HCC pathogenesis and could have great therapeutic implications [7]. Although several populations of HCC cells have been identified as CSCs based on cell surface markers, the specificity of these markers is being challenged owing to the differential expression patterns of stem-cell markers in different cell lines or patient samples [28]. Due to the lack of a generally accepted biomarker for HCC CSCs, it is reasonable to identify CSC subpopulations on the basis of functional criteria [17, 29]. Our data show that sphere-forming assays are a useful tool for enriching HCC CSCs. Indeed, tumor sphere cells exhibited CSC properties, including proliferation, self-renewal, drug resistance, and high tumorigenicity. More importantly, contrary to the cell surface marker selection strategy, which only enriches one CSC subpopulation, our data show that sphere-forming culture enriches different subpopulations of CSCs with certain HCC biomarkers, indicating that this strategy could enrich the most complete CSC population from a bulk tumor. Therefore, this HCC CSC enrichment approach could provide a deep and comprehensive understanding of HCC tumorigenesis.

Sphere-forming culture is commonly used to retrospectively confirm a certain subpopulation of tumor cells with stem characteristics [11–15]. For HCC, only two studies have reported enrichment of stem-cell subpopulations through sphere-forming culture with HCC cell lines [26, 30]. In this study, we further validated the ability of sphere-forming culture to enrich the subpopulation with stem-cell properties from HCC cell lines, and, more importantly, the ability of this culture system to enrich CSCs from fresh primary tumors. To our knowledge, this is the first study to comprehensively identify sphere cells as CSCs in freshly resected tumor specimens. More importantly, we also observed that sphere-formation rates were positively correlated with advanced malignant phenotypes, implying that tumors with advanced malignant potential are more likely to contain higher numbers of CSCs population. Thus, sphere-forming culture might be a useful way to enrich HCC CSCs, and targeting these sphere cells might be an advantageous strategy to specifically eliminate CSCs with fewer side effects. Furthermore, screening for drug sensitivity in these cells might be a promising approach to select the most specific treatment regimen for HCC patients.

The underlying mechanisms of sustaining CSC properties in HCC sphere cells was investigated, and a novel PPARα-SCD1 axis was discovered. We found that maintenance of CSC properties was regulated by PPARα pathway activation, which up-regulated SCD1 expression and induced nuclear accumulation of β-Catenin. Additionally, inhibition of the PPARα pathway or SCD1 inhibition interfered with sphere formation, and decreased the expression of CSC-related markers, resulting in loss of CSC properties. Consistent with this, one recent study demonstrated that inhibition of PPARα could interfere with sphere formation and decrease SCD1 expression, indicating the requirement of this pathway in sphere formation and CSC maintenance [31].

Recently, accumulating evidence revealed that CSCs are characterized by a high plasticity in energy substrate metabolism, and increased lipid droplet accumulation is considered as the metabolism hallmark for CSCs. In HCC, previous studies found that HCC-derived CSCs could use lipid droplets as an internal energy reservoir to foster themselves growth under hypoxic environment via an epigenetic regulatory network [32, 33]. Our results stand well in line with these ideas that lipid metabolism played a vital role in regulating CSC traits in HCC and uncovered the significance of fatty acid. As the rate-limiting enzyme in the biosynthesis of monounsaturated fatty acids from saturated fatty acids, SCD1 is overexpressed in several types of cancer [34–38]. Additionally, the livers of mouse or rats with SCD1 overexpression were susceptible to hepatocarcinogenesis [36], and SCD1 is also reported to be a biomarker for HCC aggressiveness [39, 40]. Our study indicates that the important role of SCD1 in HCC CSC maintenance occurs through regulation of the nuclear accumulation of β-Catenin, which is consistent with a recent study demonstrating that SCD1 was a vital promoter of the Wnt/β-Catenin signaling pathway [41]. Thus, targeting SCD1 could directly target the HCC stem cell subpopulation and may be a potential treatment strategy for HCC management in the future. Since LXR pathway was identified as a key metabolism regulator that rendered CSC traits for HCC cells, the crosstalk between SCD1 and LXR needs to be deeply investigated in the future.

There are some limitations of our study to be noted. First, the detailed mechanism underlying how PPARα regulates SCD1 expression remains elusive, and needs further exploration. Second, the correlation between SCD1 and other signaling pathways involved in regulating HCC stem cell phenotypes remains unknown. Additionally, although our data well demonstrated that sphere-forming cells exhibited impressive self-renew and differentiation potentials in vitro, in vivo serial serial passage assays are also needed in the future to systematically confirm the in vitro findings. These studies are ongoing in our laboratory.

## Conclusions

In summary, our data indicate that sphere-forming culture can effectively enrich the HCC CSC subpopulation, which is maintained by the PPARα-SCD1 axis. Moreover, we identified SCD1 as a key regulator of CSC properties in HCC sphere cells and suggest that targeting SCD1-related CSC machinery might provide a new insight in HCC treatment.

## Additional files

Additional file 1: **Table S1.** Primers used in present study. (DOC 54 kb)

Additional file 2: **Table S2.** Volumes of tumors of the indicated groups when mouse were sacrificed. (DOCX 12 kb)

## Data Availability

The datasets used and/or analysed during the current study available from the corresponding author on reasonable request.

## Abbreviations

5-FU: 5-Fluorouracil
ABCG2: ATP-binding cassette sub-family G member 2
APOC3: Apolipoprotein C3
BMP4: Bone morphogenetic protein 4
CCK-8: Cell Counting Kit-8
CCND1: Cyclin D1
CK19: Cytokeratin 19
CK8: Cytokeratin 8
CSC: Cancer stem cell
DMEM: Dulbecco’s modified Eagle’s medium
EpCAM: Epithelial cell adhesion molecule
FABP1: Fatty acid binding protein 1
FBS: Fetal bovine serum
FGF10: Fibroblast growth factor 10
G6P: Glucose-6-phosphatase
HCC: Hepatocellualr carcinoma
ICAM1: Intercellular adhesion molecule 1
NEDD9: Neural precursor cell expressed, developmentally down-regulated 9
N-myc: N-MYC Proto-oncogene
OCT4: Octamer binding protein 4
PCK1: Phosphoenopyruvate carboxykinase 1
PE: Phycoerythrin
PPARα: Activation of the peroxisome proliferator-activated receptor-alpha
SCD1: Stearoyl-CoA desaturase
SORBS1: Sorbin and SH3 domain containing 1
SOX2: SRY-box 2
