# Supplementary material for: Sphere-forming culture enriches liver cancer stem cells and reveals Stearoyl-CoA desaturase 1 as a potential therapeutic target
Source: BMC Cancer. 2019 Aug 1;19:760. doi: 10.1186/s12885-019-5963-z (PMC6676608; doi:10.1186/s12885-019-5963-z)
Supplement: Supplementary file 1 — Table S1. Primers used in present study. (DOC 54 kb) [file 12885_2019_5963_MOESM1_ESM.doc]

| **Table S1. Primers used in present study** | |
| --- | --- |
| Gene symbol | Sequence 5’-3’ |
| EpCAM | F:5'GAAGGCTGAGATAAAGGAGATGGG3' |
|  | R:5'TTAACGATGGAGTCCAAGTTCTGG3' |
| CD90 | F:5'TGAAGGTCCTCTACTTATCCG3' |
|  | R:5'CACGAGGTGTTCTGAGCC3' |
| CD133 | F:5'CCGCAGGAGTGAATCTTT3' |
|  | R:5'AGGACTCGTTGCTGGTGA3' |
| ABCG2 | F:5' CCGCGACAGCTTCCAATGACCT3' |
|  | R:5'GCCGAAGAGCTGCTGAGAACTGTA3' |
| CD13 | F:GCCCAAGATGTCCACGTACT |
|  | R:GAGAGATGACCCAGATCATGT |
| NEDD9 | F:TTACAAAGCCCGTGGAGAATGACA |
|  | R:AATGGCGTTGAGAAGGGAAATGAA |
| NANOG | F: ATGCCTGTGATTTGTGGGCC |
|  | R: GCCAGTTGTTTTTCTGCCAC |
| SOX9 | F:CGAAATCAACGAGAAACTGGAC |
|  | R: ATTTAGCACACTGATCACACG |
| UGT1A1 | F:GCAGCAGAGGGGACATGAAATAGT |
|  | R:GGGAACAGCCAGACAAAAGCATAG |
| UGT1A9 | F:TGGAAAGCACAAGTACGAAGTA |
|  | R:CTTCTTCAAGATAGTGGCAAAG |
| CD73 | F:GATCGAGCCACTCCTCAAA |
|  | R:GCCCATCAGAAGTGAC |
| SCD | F:TCTTGTCCCTATCGCCCACTGCAG |
|  | R:AGCTCAGAGCGCGTGTTCAA |
| S100A14 | F:TGGAAAGCACAAGTACGAAGTA |
|  | R:CTTCTTCAAGATAGTGGCAAAG |
| VIL1 | F:CATCGGCGAGAAGCAGCATTAC |
|  | R:GCCCGGTCTCCAAGTTGTTAGTTC |
| S100A9 | F:CGAAATCAACGAGAAACTGGAC |
|  | R: ATTTAGCACACTGATCACACG |
| EFNA1 | F:CCCACGCCTCTTCCCACTTG |
|  | R:TACTGACCCGTTTTGAGGCTGCTA |
| EFNA2 | F:TCCAGCTCTTCACGCCCTTCT |
|  | R:CCGCACGTACACCTTCAGTCG |
| MDM4 | F:CCATTTCGGCTCCTGTCGTTAG |
|  | R:GTTCCCGTCTCGTGGTCTTTTCT |
| CCNG1 | F:CACACGATAATGGCCTCAGAATG |
|  | R:CCAAATAAAAGCAGCTCAGTCCA |
| THBS1 | F:GCGTGGCCAATGCGACTTA |
|  | R:TCTGCCTGATCTGGGTTGTGG |
| GTSE1 | F:CCAAGCGGGTCGATGTTTCT |
|  | R:CTGTCCGGGGAGTCACCAATA |
| FABP1 | F:AGCCCCCTATAAAACAGCCTACA |
|  | R:CAGCGGTGATGGTGAACTTGA |
| PPARα | F:TTGTGGCTGCTATCATTTGCTGTG |
|  | R:CTGCCGGAGGTCTGCCATTT |
| APOC3 | F:GCCCCGGGTACTCCTTGTTG |
|  | R:CATCCTTGGCGGTCTTGGTG |
| PCK1 | F:AGCCCCCTATAAAACAGCCTACA |
|  | R:CAGCGGTGATGGTGAACTTGA |
| PDK1 | F:TGAACTGACCTTGCCACAT |
|  | R:TGAAGCAGCACTGAACACG |
| SORBS1 | F:AAACCCAGTAAGAGTAAGACC |
|  | R:TAGCCACTACAGTCCACAAT |
| β-actin | F:5'TTGTTACAGGAAGTCCCTTGCC3' |
|  | R:5'ATGCTATCACCTCCCCTGTGTG3' |
|  |  |
