# Supplementary material for: Sphere-forming culture enriches liver cancer stem cells and reveals Stearoyl-CoA desaturase 1 as a potential therapeutic target
Source: BMC Cancer. 2019 Aug 1;19:760. doi: 10.1186/s12885-019-5963-z (PMC6676608; doi:10.1186/s12885-019-5963-z)
Supplement: Supplementary file 2 — Table S2. Volumes of tumors of the indicated groups when mouse were sacrificed. (DOCX 12 kb) [file 12885_2019_5963_MOESM2_ESM.docx]

| **Volumes of tumors of the indicated groups when mouse were sacrificed** | | | | | | |
| --- | --- | --- | --- | --- | --- | --- |
| Phenotype | Tumor volumes (mm^3^) | | | | | |
| 5×10^2^ Sphere-forming Huh7 cells | 1526.08 | 1068.18 | 1017.94 | 1310.33 | N.A. | N.A. |
| 1×10^3^ Sphere-forming Huh7 cells | 2016.78 | 2328.71 | 1293.27 | 2585.52 | 1669.96 | 1580.29 |
| 2×10^2^ Sphere-forming Primary cells | 785.67 | 1323.57 | 1058.83 | N.A. | N.A. | N.A. |
| 5×10^2^ Sphere-forming Primary cells | 982.09 | 1194.02 | 1868.87 | 1328.95 | N.A. | N.A. |
| 1×10^3^ Sphere-forming Primary cells | 1217.56 | 1707.76 | 2338.95 | N.A. | N.A. | N.A. |
| 5×10^3^ Sphere-forming Primary cells | 2008.36 | 1610.95 | 2537.67 | 1853.38 | 1557.04 | N.A. |
